# Immunotherapy in Advanced Cutaneous Melanoma: From the Optimal Treatment Duration to the Impact on Survival in Case of Early Discontinuation Due to Immune-Related Adverse Events

**DOI:** 10.3390/biom15050651

**Published:** 2025-04-30

**Authors:** Giacomo Triggiano, Gaetano Pezzicoli, Marco Tucci

**Affiliations:** 1 Medical Oncology Unit, Policlinico of Bari, 70124 Bari, Italy; triggianogiacomo@gmail.com (G.T.);; 2 Department of Interdisciplinary Medicine (DIM), University of Bari ‘Aldo Moro’, 70124 Bari, Italy

**Keywords:** cutaneous melanoma, immune checkpoint inhibitors, immune-related adverse events, anti-PD1, combo-immunotherapy, treatment discontinuation

## Abstract

Immune checkpoint inhibitors (ICIs) have improved the prognosis of patients with cutaneous melanoma. Immunotherapy (IT) is generally well tolerated, but an increasing area of investigation concerns the optimal treatment duration of anti-programmed cell death-1 (anti-PD1) regimens to limit the immune-related adverse events in patients who obtained a clinical response. Another point of interest is the impact of the early discontinuation of ICIs on the maintenance of response in terms of survival in patients developing grade 3–4 adverse events that mostly occur in those receiving the combo-IT. Currently, we are still far from having final conclusions on these topics and, thus, the present review aims to describe the recent data about the optimal treatment duration and the maintenance of response in the case of early discontinuation. In this context, we include data on the real life of patients from our Medical Oncology Center who discontinued anti-PD1 after at least a stable disease or those interrupting the combo-IT due to adverse events.

## 1. Introduction

Cutaneous melanoma (CM) accounts for almost 90% of skin cancer-related deaths due to its high metastatic potential, with the incidence rate increasing, particularly in Caucasians [1,2,3]. The advent of immune checkpoint inhibitors (ICIs) and BRAF inhibitors has markedly improved the prognosis of patients with CM, especially considering that the estimated life expectancy prior to their introduction was approximately 6 months [4,5,6]. In this context, significant progress has been achieved in patients with unresectable or metastatic CM who received ICIs as single agents or in combination [7,8,9].

Although immunotherapy (IT) is better tolerated as compared to chemotherapy [10,11,12], the optimal treatment duration with first-line anti-PD1 agents in responding patients is mostly based on the long-lasting effects observed after treatment disruption, aiming to minimize immune-related adverse events (irAEs) [13,14].

Many randomized clinical trials using anti-PD1 monoclonal antibodies (MoAb), namely pembrolizumab, continued treatment up to 2 years, while nivolumab was administered beyond 24 months. Therefore, the prevalent data derive from pembrolizumab-based regimens [15].

However, the 2025 National Comprehensive Cancer Network (NCCN) guidelines state that 2 years of treatment is the standard of care for patients with at least stable disease (SD), regardless of BRAF mutation status, prior BRAF inhibitor use, or poor prognostic features such as elevated lactate dehydrogenase (LDH) levels and the presence of brain metastases [16]. This strategy helps to limit the risk of irAEs, which is strongly associated with treatment duration [17] and is more frequent with combination therapy as compared to single agents [18,19,20]. In fact, the higher rate of irAEs is mainly attributed to the addition of ipilimumab and typically occurs within the first 6 months of treatment [21], thus resulting in premature discontinuation in 50% of cases and prolonged immunosuppressive therapy [22,23]. Nevertheless, limited real-world data are available regarding the maintenance of response in terms of survival in patients who discontinue therapy due to irAEs.

This review aims to provide an overview of current data concerning the optimal treatment duration in patients receiving first-line therapy with anti-PD1 MoAbs and treatment efficacy in terms of survival in cases of early interruption due to irAEs. Moreover, we summarize the main ongoing trials addressing these critical points, alongside other recent studies evaluating alternative schedules optimized to limit adverse effects without restraining therapeutic benefit. Lastly, we present retrospective real-world data from our oncology unit, describing the duration of maintenance response in patients who discontinued IT with anti-PD1 MoAbs, regardless of disease progression, as well as in those who interrupted combo-IT for irAEs.

## 2. The Anti-PD1-Based Regimen in the First-Line Setting

A pioneering study with the anti-Cytotoxic T-Lymphocyte Antigen 4 (CTLA-4) agent ipilimumab reshaped the treatment of metastatic melanoma, effectively limiting the use of chemotherapy due to its poor effectiveness [24]. However, ipilimumab is currently not considered the best option in the upfront first-line strategy because data from KEYNOTE-006 [25], which compared ipilimumab with pembrolizumab, demonstrated the superiority of the anti-PD1 agent in terms of progression-free survival (PFS) and overall survival (OS). The 10-year follow-up showed a median OS of 32.7 months in the pembrolizumab arm compared to 15.9 months in the ipilimumab arm [HR, 0.71]. Median PFS was 9.4 and 3.8 months, respectively [HR, 0.64].

In addition, the anti-PD1 MoAb nivolumab received FDA approval as a result of similar results in a phase-3 study, with a 1-year OS rate of 72.9% and a median PFS of 5.1 months [26].

Based on the outstanding results of the CheckMate 067 phase-3 trial, the current standard of care for metastatic and unresectable melanoma is a combination of nivolumab and ipilimumab, which is the treatment of choice for patients with unfavourable prognostic factors, such as elevated LDH levels, rapidly progressive disease, and brain metastases [27,28]. The 10-year update demonstrated sustained long-term survival with the combination, with a median OS of 72 months (the longest survival reported in any phase-3 study with anti–PD1 agents for any cancer type) [20].

However, an innovative combination of ICIs like relatlimab and nivolumab was recently approved. Relatlimab is a first-in-class inhibitor of Lymphocyte-Activation Gene 3 (LAG-3), a surface-expressed molecule that downregulates T-lymphocyte proliferation. After a median follow-up of 33.8 months, the phase 2/3 RELATIVITY-047 study showed the superiority of relatlimab/nivolumab over nivolumab alone, with a median PFS of 10.2 versus 4.6 months. Moreover, the median OS was 51.0 months and 34.1 months, respectively. Interestingly, this benefit was associated with a consistent safety profile, with 22% of patients experiencing grade 3–4 irAEs, leading to 10% of discontinuation. This strongly contrasts with the higher risk observed with the ipilimumab–nivolumab combination (63% and 34%, respectively) [29].

The relevant clinical trials using anti-PD1 agents are listed in Table 1.

## 3. Landscape of Treatment Discontinuation

### 3.1. Anti-PD1 Monoclonal Antibodies

The phase I KEYNOTE-001 clinical trial partly addressed the topic of discontinuation following a protocol amendment that included 67 patients showing complete response (CR) after more than 6 months of pembrolizumab. They completed a minimum of two courses of therapy after the confirmed CR, showing 24-month disease-free survival (DFS) from the time of CR of 90%. Real-life data confirmed that relapse occurs more frequently in patients who received less than 6 months of an anti-PD1 MoAb [30,31].

Relevant evidence comes from the KEYNOTE-006 trial and its extension, KEYNOTE-587, whose 10-year follow-up update showed an OS rate of 34.0% for patients receiving pembrolizumab. Moreover, the 8-year OS rate based on best objective response (OR) was 78.1% among patients with CR, 58.7% for partial response (PR), and 21.8% in SD, suggesting a correlation between the quality of response and survival [25].

Jansen et al. studied the outcome of 185 patients who discontined anti-PD1 MoAb without evidence of progressive disease (PD) or treatment-limiting toxicities (TLTs). The median treatment duration (mTD) was 12 months, considerably shorter compared to the maximum treatment duration of 24 months in the KEYNOTE-006 trial. The best OR at the time of discontinuation was CR in 63%, PR in 24%, and SD in 9% of patients, respectively. After a median follow-up of 18 months post-treatment, 78% of patients were free of progression. The median time to progression was 12 months and was less frequent in patients with CR (14%) compared to PR (32%) and SD (50%). Moreover, in patients achieving a CR, the risk of relapse after discontinuation was lower when treated for more than 6 months [32].

Warner et al.’s retrospective study analyzed 396 patients who discontinued anti-PD1 treatment and had at least 3 months of follow-up after discontinuation. After a median follow-up of 21 months from the time of CR and a median TD of 7 months, the 3-year OS was 72.1%, with no significant correlation between the duration of treatment and risk of relapse [33].

A study by Valentin et al. investigated the PFS in patients who stopped anti-PD1 MoAbs for OR or TLTs. Out of 65 patients, 38% discontinued for CR and 43% for TLTs. After a median follow-up of 15.7 months from discontinuation, only 18% of patients relapsed, while the median PFS was not reached, suggesting a long-lasting response regardless of the reason for discontinuation [34].

An observational trial conducted by the Italian Melanoma Intergroup retrospectively assessed the risk of relapse in 237 patients who discontinued anti-PD1 therapy due to CR, TLTs, or personal decision, after a mTD of 33 months. Specifically, 54% of patients stopped treatment due to CR, 31% due to adverse events (37 in CR, 27 in PR, and 10 in SD), and 14.8% by their own choice (12 patients in CR, 17 in PR, and 6 in SD). After a median follow-up of 21 months, PFS was 85.7%, while 34 patients developed disease progression after a median of 12 months. Specifically, 29% of relapses occurred during CR, 50% after discontinuation for toxicity (7 in CR, 5 in PR, 5 in SD), and 20% in those who discontinued by their own decision. Thus, in 70.6% of cases, recurrence was observed in patients who had not achieved at least CR at the time of discontinuation [35].

Furthermore, Swami’s real-life study investigated the interruption of anti-PD1 due to irAEs in 16 patients with advanced melanoma, showing durable clinical benefit in 81% of them, with a median PFS of 24 months and median OS not reached, suggesting clinical efficacy despite the risk of TLTs [36]. Lastly, a further retrospective study explored patients who discontinued by choice without active disease (24%) after a median TD of 12 months and those who discontinued due to irAEs after a median TD of 4 months. The 3-year event-free survival (EFS) rate was 95% and 71%, respectively, suggesting an encouraging benefit, especially in the first group [37].

In conclusion, despite the heterogeneity of the above-mentioned data, the long-lasting effect of anti-PD1 therapy seems not to be exclusively guaranteed by the treatment duration but also by the quality of the response. On the contrary, it is not particularly restrained by irAEs.

### 3.2. Combination Therapy with Anti-PD1 and CTLA-4 MoAbs

The astonishing results in terms of OS documented in the 10-year update of CheckMate 067 are, however, associated with a high incidence of grade 3–4 irAEs, which cause discontinuation even before the completion of the induction phase. Any grade of toxicity occurs in 96% of patients, with 63% experiencing grade 3 or 4 events (34% leading to discontinuation), but it does not negatively impact survival. In fact, the melanoma-specific survival (MSS) rate in case of discontinuation for toxicity was higher than in the intention-to-treat (ITT) population: to date, 54% vs. 52% with the combination, 71% vs. 44% with nivolumab, and 29% vs. 23% with ipilimumab [19].

A post hoc retrospective analysis from the CheckMate 067 and CheckMate 069 trials evaluated the efficacy of the combination therapy in cases of interruption for irAEs during the induction phase. Among 407 patients who received the combination, 43% discontinued treatment due to any AE, while the others stopped for other reasons, including disease progression. After a minimum follow-up of 18 months, median PFS was 8.4 months for patients discontinuing due to irAEs, compared to 10.8 months for the other group. Median OS was not reached for either group. The objective response rate (ORR) was 58% for the first cohort and 50% for patients who did not stop [38].

The multicenter retrospective study by Dimitriou et al. [39] evaluated the outcome of 125 patients with or without brain disease after elective treatment discontinuation from anti-PD1 monotherapy (n = 97) or combined with anti-CTLA-4 (n = 28) due to CR (group A, n = 86) or TLT/investigator’s decision (n = 33 and 6, respectively, group B) with subsequent CR. Many patients (56%) who discontinued treatment due to TLT received combo-IT. In group A, after an mTD of 22 months and a median time to CR of 9 months, seven patients experienced a recurrence: two (1.6%) were treated with combo-IT and five (4%) with anti-PD1 alone. In group B, after a median TD of 3 months and a median time to CR of 7 months, three patients had disease recurrence (2.4%) (one treated with combo-IT and two with single anti-PD1).

Recently, Amiot et al. conducted an open-label series investigating the effect of discontinuation at specific timepoints among patients with SD, although excluding those suffering from TLTs or treated for less than 6 months. Among 1017 participants, only 435 were finally enrolled at the 6-month discontinuation timepoint (221 treated with nivolumab–ipilimumab, 475 with pembrolizumab, and 321 with nivolumab), mainly due to early discontinuation for toxicity and disease progression. The results after a 6-month break in treatment indicated that individuals who stopped their treatment had a lower overall survival (OS) compared to those who continued their treatment for at least three months. Over a 48-month period, the survival difference was 37.8%, corresponding to a difference in survival time of 8.3 months. The 12-month and 18-month discontinuation results showed that stopping treatment yielded a 48-month survival that was, respectively, 3.7% and 4.2% higher than continuing treatment, with a 48-month survival difference of 0.7 and 0.4 months, respectively. The 24-month discontinuation results favored discontinuing treatment over continuing at that decision point, with an absolute 48-month survival difference of 10% higher and a 48-month survival one month higher. This suggests that stopping ICIs before 6 months and for more than two years might be discouraged [3].

### 3.3. Alternative Strategies

Based on the clinical benefit provided by combo-IT, recent studies have attempted alternative schedules to avoid discontinuation due to adverse events. To date, in the phase IIIb/IV CheckMate 511 Trial, 360 patients were randomly assigned 1:1 to nivolumab 1 mg/kg (NIVO1) + ipilimumab 3 mg/kg (IPI3) versus nivolumab 3 mg/kg (NIVO3) + ipilimumab 1 mg/kg (IPI1) once every 3 weeks for four doses, followed by maintenance with nivolumab 480 mg once every 4 weeks until disease progression or unacceptable toxicity. At a minimum follow-up of 12 months, the incidence of treatment-related grade 3 to 5 AEs was higher in patients receiving ipilimumab at 3 mg/kg (48% vs. 34%), with similar results in terms of the ORR rate and median PFS, while median OS was not reached [40]. Therefore, reducing or reversing the dosage of combo-IT might provide adequate efficacy with limited adverse effects.

The phase 1b KEYNOTE-029 study explored the combination of standard pembrolizumab with alternative doses of ipilimumab (1 mg/kg every 3 weeks for four cycles; 50 mg every 6 weeks for four doses; and 100 mg every 12 weeks for four doses). The results showed treatment-related grade 3–5 AE rates of 47%, 24%, and 39%, respectively, with similar ORRs compared to the standard dose of the ipilimumab/nivolumab regimen [41,42]. However, none of these alternative schedules have been included in clinical practice due to the small sample sizes and the lack of long-term data.

## 4. Our Monocentric Experience

### 4.1. Patients and Methods

Herein, we describe real-life and retrospective data of metastatic melanoma patients treated at the Medical Oncology Unit of the University Hospital of Bari. We examined the efficacy in terms of maintenance of response in two different cohorts of patients that currently represent distinct scenarios in clinical practice: those who discontinued anti-PD1 MoAb administered as single agents regardless of disease progression, and those who interrupted combo-IT early exclusively due to irAEs.

The purpose of differentiating these two subgroups arises from the need to evaluate their distinct survival outcomes, especially in relation to the forced discontinuation of combo-IT, for which real-life data are extremely limited. In this context, for both groups, demographic data were collected, including age, gender, and ECOG Performance Status. Tumor-specific characteristics included both primary and metastatic sites classified according to the AJCC 8th edition (M1a-d), BRAF and NRAS status, LDH baseline levels, and previous adjuvant treatments. Reported toxicities were evaluated according to the CTCAE (Common Terminology Criteria for Adverse Events) classification v5.0, with grades 3–4 leading to discontinuation [43]. As shown in Table 2, the best objective response (OR) was evaluated according to immune-related response criteria [iRECIST (Immunotherapy Response Evaluation Criteria in Solid Tumours)] and divided into CR, PR, SD, and PD using CT scan or 18FDG-PET/CT [44].

In examining treatment duration, we investigated the efficacy outcomes of both cohorts in terms of PFS, OS, therapy-free survival (TFS), and time to treatment response (TTR). The observation period started in January 2017 and continued until October 2024. Treatment duration was the time between the date of the first and the last dose of IT. PFS was defined as the time between the date of the first dose of systemic therapy and that of radiologically or clinically confirmed progression, leading to the initiation of a second-line systemic treatment or the last-known alive/last follow-up date. Thus, oligoprogression treated with locoregional procedures was not considered. OS was defined as the time between the diagnosis of melanoma and the date of either death, last known alive, or last follow-up date. TFS was calculated as the time between the last dose of IT and disease progression, leading to second-line systemic therapy or the last follow-up date. TTR was defined as the time between the first dose of systemic therapy and the first clinical or radiological response per iRECIST criteria obtained.

Descriptive statistics were used to summarize patient characteristics and treatment-related variables. Survival and response outcomes were visualized using Swimmer Plots realized with R Studio software (R version 4.3.1).

### 4.2. Results

**Figure 1:**
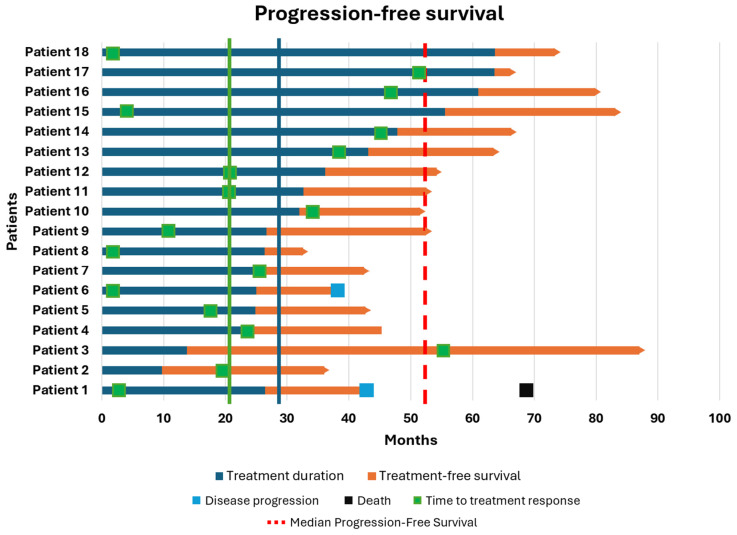
Swimmer plot showing the outcomes of the first cohort of patients enrolled in our medical unit, namely those who discontinued immunotherapy with anti-PD1 single agents independently of disease progression.

In the first cohort, 18 patients who discontinued an anti-PD1 MoAb were enrolled. The best OR at the time of discontinuation was CR in 11 patients (61.2%), PR in 6 patients (33.3%), and SD in 1 patient (5.5%). After a median follow-up of 19.2 months, the mTD was 29.3 months (range: 9.7–63.6). As shown in Figure 1, the median PFS was 52.5 months, although this was not consistent, as only two patients developed progression after discontinuation (at 42.9 and 38.0 months, respectively), while 88.8% of patients maintained their response. The median OS was 83.3 months (range: 36.5–206.8), with only one patient dying from cancer-related causes (OS: 69.56 months). The median TFS was 18.8 months (range: 2.53–46.16), and the median TTR was 20.0 months (range: 1.46–54.53). Both patients who progressed after anti-PD1 therapy had the same baseline characteristics: BRAF and NRAS wild-type, regular LDH levels, M1b disease, and CR as the best OR. Clearly, given the small sample size, it is not possible to draw meaningful conclusions from sub-analyses regarding the prognostic impact of these factors on survival.

**Figure 2:**
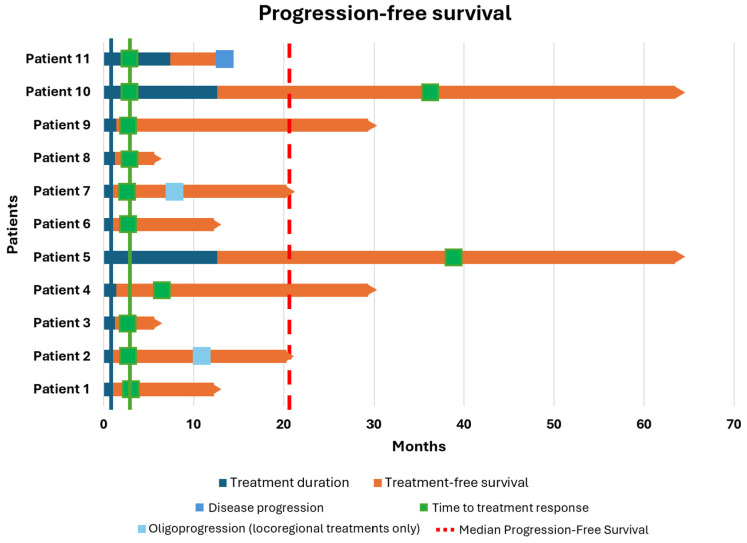
Swimmer plot presenting the outcomes of the second cohort of patients, namely those who interrupted combo-immunotherapy with nivolumab–ipilimumab early due to irAEs under a controlled disease.

The second cohort consisted of 11 patients who interrupted combo-IT due to TLTs. Overall, except for one patient, all interrupted treatment during the induction phase. As shown in Table 2 and based on the American Joint Committee on Cancer (AJCC) 8th edition, 73% of patients had brain metastases, and 27% had PD-L1 levels less than 1%. The best OR was CR in four patients (36.4%), PR in four patients (36.4%), and SD in three patients (27.2%). After a median follow-up of 19.4 months (range: 4.4–50.8), the median TD was 1.2 months (range: 0.7–12.6). As shown in Figure 2, the median PFS was 20.3 months, although this was not consistent, as only one patient experienced progression. The median OS was 60.7 months (range: 6.6–96.6), with all patients still alive. The median TFS was 19.4 months (range: 4.4–50.8), and the median TTR was 2.9 months (range: 2.4–39.3). Considering negative prognostic factors, six out of eight patients with M1d asymptomatic disease did not experience encephalic recurrence, with a median TD of 1 month. Only two patients developed encephalic oligoprogression (at 7 and 11 months after discontinuation of therapy). In both cases, they were treated with stereotactic radiotherapy (RT) without undergoing further systemic therapies and were still free of disease at the time of the follow-up closure.

### 4.3. Toxicity

As per the study objective, TLTs were assessed exclusively in the second cohort of patients, finding that all of them interrupted combo-IT due to grade 4 adverse events. Specifically, hepatic toxicity occurred in six patients (54.5%), gastrointestinal toxicity in two patients (18.2%), and one case each of hypothyroidism, hyperthyroidism, and hypophysitis was registered. These adverse events were managed and completely resolved through the prolonged use of specific doses of steroid-based immunosuppressive therapy, except for thyroid toxicity, which was treated with targeted endocrinological treatment. Steroid therapy was permanently discontinued without any iatrogenic sequelae (e.g., diabetes, osteonecrosis), while thyroid replacement therapy was continued on a chronic basis. Although the sample size is limited, these findings support the hypothesis that prolonged steroid therapy does not compromise the efficacy of combo-IT.

### 4.4. The Role of RT in the Management of Oligoprogression

Oligoprogressive disease is frequently encountered in CM, with the brain being the most involved organ, although extracranial sites may also be affected. In this context, a frequent adopted strategy is the integration of locoregional approaches for controlling the disease progression and deferring the initiation of subsequent lines of therapy.

Although melanoma is known as a radioresistant cancer due to its intrinsic DNA repair competence for sublethal damage, radiotherapy (RT) remains the most widely employed strategy to achieve this result and is associated with the greatest clinical benefit. To date, several studies have focused on defining the optimal timing for combining RT with IT. Indeed, RT can be successfully applied to different target organs in two settings: after disease progression (Post-Escape Radiotherapy, PER) or during the induction phase (Peri-Induction Radiotherapy, PIR) to enhance the proinflammatory microenvironment of the tumor. Regarding PIR, there are insufficient data to define the optimal timing for starting IT. Furthermore, the available studies are mostly retrospective and involve only single-agent IT regimens [45]. On the other hand, PER is more commonly adopted in clinical settings, particularly in the management of brain metastases. Referring to our real-life data, encephalic involvement was the only site of oligoprogression among our patients and was effectively treated with stereotactic PER, which is widely adopted as an integrative procedure to control the intracranial response, reduce symptoms, and avoid changes in systemic treatment [46,47]. Moreover, RT has proven to be a safe procedure that does not negatively impact the patients’ quality of life nor exacerbate the toxicity profile of ongoing IT [48].

## 5. Future Perspectives

Ongoing clinical trials, such as STOP-GAP, DANTE, SAFE STOP, and SAFE STOP IPI-NIVO (Table 3), are evaluating the elective interruption of anti-PD1-based regimens in metastatic melanoma patients, but preliminary results are not yet available [49,50,51].

The Canadian STOP-GAP trial is a randomized phase 3 study aimed at determining the impact on survival in 614 estimated melanoma patients treated with an anti-PD1 agent, either continuously (non-stop) or on an intermittent schedule (taking breaks). The goal is to minimize irAEs, healthcare system costs, and improve quality of life.

The DANTE trial is a randomized, non-blinded, non-inferiority phase III study recruiting 1208 patients who have received one year of first-line therapy with single-agent anti-PD1, with or without CTLA-4 inhibitors. These patients are randomly assigned 1:1 to either continue treatment (until disease progression or unacceptable toxicity, or at least 2 years in the absence of progression) or stop treatment. The primary objective is to determine if continuing treatment beyond one year is necessary.

The SAFE STOP Dutch prospective single-arm trial includes 200 patients undergoing early discontinuation of first-line monotherapy with nivolumab or pembrolizumab after achieving a confirmed CR or PR. The primary endpoint is to compare the rate of ongoing response 24 months after discontinuation with respect to patients who have completed two years of therapy.

The SAFE STOP IPI-NIVO trial is an ongoing prospective single-arm study in the Netherlands enrolling 80 patients treated with the nivolumab–ipilimumab combination. These patients will undergo early discontinuation of maintenance therapy after achieving a confirmed CR or PR. The aim is to assess whether early discontinuation after four induction cycles is safe in terms of efficacy and to reduce the risk of irAEs.

Preliminary results are eagerly awaited, as they may offer critical insights into determining the optimal treatment duration for anti-PD1-based regimens and the feasibility of alternative schedules with equivalent efficacy and reduced healthcare costs. Additionally, prospective data from the SAFE STOP IPI-NIVO trial will provide valuable guidance on the management of TLTs from a preventive perspective.

## 6. Conclusions

ICIs have improved the prognosis of patients with metastatic melanoma, although this advantage can currently be recorded in various tumors; however, the prolonged use of these drugs may expose patients to possible adverse events, restraining quality of life with an impact on healthcare costs [18].

In this review, we reported the current evidence about two unmet needs in advanced melanoma: the optimal treatment duration of the anti-PD1-based regimens for responding patients and the durability of the response in terms of survival for patients discontinuing the therapy due to irAEs.

Regarding the first topic, international guidelines suggest 2 years as the optimal period, but there is not a consensus in this area. However, according to the large amount of retrospective data examined, this period seems to be a good compromise that requires further confirmation based on the best OR and/or any interruptions due to toxicity.

Regarding the second topic, there are still few data about survival in patients who have discontinued due to TLTs in the absence of disease progression, although the efficacy is apparently guaranteed, even in the case of early interruption.

In conclusion, our real-life data retrospectively attempted to contribute to these topics, thus confirming the long-lasting effect of ICIs for both reasons of discontinuation. However, further data from randomized perspective trials are urgently needed to establish stronger guidelines regarding the optimal treatment duration for first-line advanced melanoma therapy. Additionally, more real-life evidence is still required to confirm the long-term effects on survival in patients requiring early discontinuation due to toxicity.

## Figures and Tables

**Table 1:** Overview of the anti-PD1-based regimens currently used in the first-line setting.

Trial	Phase	Setting	ICI’s	Last Update	OS (Months)	PFS (Months)
KEYNOTE 006	III	III unresectable/IV	pembrolizumab vs. ipilimumab	2024 (10 year)	32.7 vs. 15.9	9.4 vs. 3.8
CheckMate 066	III	III unresectable/IV	nivolumab vs. dacarbazine	2020 (1 year)	37.3 vs. 11.2	5.1 vs. 2.2
CheckMate 067	III	III unresectable/IV	nivolumab + ipilimumab vs. nivolumab vs. ipilimumab	2024 (10 year)	71.9 vs. 36.9 vs. 19.9	28% vs. 21% vs. 16% *
Relativity 047	II/III	III unresectable/IV	nivolumab + relatlimab vs. nivolumab	2024 (3 year)	51 vs. 34.1	10.2 vs. 4.6

ICI’s, immune checkpoint inhibitors. OS, overall survival. PFS, progression-free survival. * data available in terms of response rate.

**Table 2:** Baseline patients’ characteristics.

Characteristics	Cohort 1 (Anti PD-1)	Cohort 2 (Anti PD-1 + Anti CTLA-4)
**Number of patients**	18	11
**Median Age/range**	68.4 (28–85)	65.8 (44–68)
**Gender**		
Male	13 (72.2%)	9 (81.8%)
Female	5 (27.8%)	2 (18.2%)
**ECOG**		
0	16 (88.8%)	9 (81.8%)
1+	2 (11.2%)	2 (18.2%)
**Stage**		
M1a	5 (27.8%)	
M1b	2 (11.1%)	
M1c	9 (50%)	3 (27.3%)
M1d	2 (11.1%)	**8 (72.7%)**
**BRAF mutated**	3 (16%)	8 (72.7%)
**Prior Adjuvant Treatment**	3 (16%) [2 TT + 1 intron]	4 (36.4%) [TT]
**Elevated LDH**	5 (27.8%)	2 (18.2%)
**NRAS mutated**	6 (33%)	0
**Best objective response**		
Complete Response **(CR)**	11 (66.2%)	4 (36.4%)
Partial Response **(PR)**	6 (33.3%)	4 (36.4%)
Stable Disease **(SD)**	1 (5.5%)	3 (27.2%)

**Table 3:** Overview of the ongoing trials evaluating the discontinuation of the anti-PD1 MoAb-based regimens.

Trial (NCT Number)	Identifier	Phase	Status	Setting	Design
STOP-GAP	NCT02821013	III	Recruiting	III unresectable/IV	Single anti-PD1 agent given continuously (non-stop) vs. intermittent schedule (taking breaks)
DANTE	EDURACT2017–002435–42	III	Completed	III unresectable/IV	Anti-PD1-based regimen given for 1 year vs. beyond progression
SAFE STOP	NL7293	Not applicable	Recruiting	III unresectable/IV	Rate of ongoing responses at 24 months after discontinuation of PD1 blockade
SAFE STOP IPI-NIVO	NCT05652673	Not applicable	Recruiting	III unresectable/IV	Efficacy of early discontinuation under maintenance therapy with nivolumab after ipilimumab–nivolumab

Anti-PD1, anti-programmed cell death 1.

## Data Availability

Not applicable.

## Abbreviations

The following abbreviations are used in this manuscript:

CM	Cutaneous Melanoma
ICIs	Immune checkpoint inhibitors
Anti-PD1	Anti-programmed cell death 1
IT	Immunotherapy
irAEs	Immune-related adverse events
LDH	Lactate dehydrogenase
Mo	Months
moAb	Monoclonal antibody
CTLA-4	Cytotoxic T-Lymphocyte Antigen 4
PFS	Progression free survival
OS	Overall survival
LAG-3	Lymphocyte-Activation Gene 3
CR	Complete response
DFS	Disease free survival
OR	Objective response
PR	Partial response
SD	Stable disease
PD	Progression disease
TLTs	Treatment limiting toxicities
mTD	Median treatment duration
ITT	Intention to treat
MSS	Melanoma specific survival
AJCC	American Joint Committee on Cancer
TFS	Treatment free survival
TTR	Time to treatment response
RT	Radiotherapy
PER	Post-Escape Radiotherapy
PIR	Peri-Induction Radiotherapy

## References

[1] Long G.V., Swetter S.M., Menzies A.M., Gershenwald J.E., Scolyer R.A. (2023). Cutaneous melanoma. Lancet.

[2] Adler N.R., Haydon A., McLean C.A., Kelly J.W., Mar V.J. (2017). Metastatic pathways in patients with cutaneous melanoma. Pigment. Cell Melanoma Res..

[3] Amiot M., Mortier L., Dalle S., Dereure O., Dalac S., Dutriaux S.C., Leccia M.T., Maubec E., Arnault J.P., Brunet-Possenti F. (2024). When to stop immunotherapy for advanced melanoma: The emulated target trials. eClinicalMedicine.

[4] Lazaroff J., Bolotin D. (2023). Targeted Therapy and Immunotherapy in Melanoma. Dermatol. Clin..

[5] Luke J.J., Flaherty K.T., Ribas A., Long G.V. (2017). Targeted agents and immunotherapies: Optimizing outcomes in melanoma. Nat. Rev. Clin. Oncol..

[6] Garbe C., Eigentler T.K., Keilholz U., Hauschild A., Kirkwood J.M. (2011). Systematic review of medical treatment in melanoma: Current status and future prospects. Oncologist.

[7] Farkona S., Diamandis E.P., Blasutig I.M. (2016). Cancer immunotherapy: The beginning of the end of cancer?. BMC Med..

[8] Robert C., Schachter J., Long G.V., Arance A., Grob J.J., Mortier L., Daud A., Carlino M.S., McNeil C., Lotem M. (2015). Pembrolizumab versus Ipilimumab in Advanced Melanoma. N. Engl. J. Med..

[9] Dimitrova M., Weber J. (2024). Melanoma-Modern Treatment for Metastatic Melanoma. Cancer J..

[10] Mateus V.D., Ferrari M., Zaleski T., Vilas Boas R.R., Ribeiro E.R., Vaz R.S., Cavassin F.B. (2021). Chemotherapy in focus: A meta-analysis confronts immunotherapy in the treatment of advanced melanoma. Crit. Rev. Oncol. Hematol..

[11] Nishijima T.F., Shachar S.S., Nyrop K.A., Muss H.B. (2017). Safety and tolerability of PD-1/PD-L1 inhibitors compared with che motherapy in patients with advanced cancer: A meta-analysis. Oncologist.

[12] Boutros A., Bruzzone M., Tanda E.T., Ceppi M., Lambertini M. (2021). Health-related quality of life in cancer patients treated with immune checkpoint in hibitors in randomised controlled trials: A systematic review and meta-analysis. Eur. J. Cancer.

[13] De Risi I., Sciacovelli A.M., Guida M. (2022). Checkpoint inhibitors immunotherapy in metastatic melanoma: When to stop treatment?. Biomedicines.

[14] Bogani G., Cinquini M., Signorelli D., Pizzutilo E.G., Romanò R., Bersanelli M., Raggi D., Alfieri S., Buti S., Bertolini F. (2023). A systematic review and meta-analysis on the optimal treatment duration of checkpoint inhibitors in solid tumors: The OTHERS study. Crit. Rev. Oncol. Hematol..

[15] Keilholz U., Ascierto P.A., Dummer R., Robert C., Lorigan P., Van Akkooi A., Arance A., Blank C.U., Chiarion Sileni V., Donia M. (2020). ESMO consensus conference recommendations on the management of metastatic melanoma: Under the auspices of the ESMO Guidelines Committee. Ann. Oncol..

[16] NCCN Clinical Practice Guidelines in Oncology (NCCN Guidelines) Melanoma: Cutaneous Version 1.2025—December 20, 2024. https://www.lcgdbzz.org/custom/news/id/5c1b6ade-0ad7-4566-924a-b278ee38ac47.

[17] Postow M.A., Sidlow R., Hellmann M.D. (2018). Immune-related adverse events associated with immune checkpoint blockade. N. Engl. J. Med..

[18] Liu K., Wang Y.H., Luo N., Gong J., Wang J., Chen B. (2023). Treatment-related gastrointestinal adverse events of nivolumab plus ipilimumab in randomized clinical trials: A systematic review and meta-analysis. Future Oncol..

[19] Wolchok J.D., Chiarion-Sileni V., Rutkowski P., Cowey C.L., Schadendorf D., Wagstaff J., Queirolo P., Dummer R., Butler O.M., Hill A.G. (2025). Final, 10-Year Outcomes with Nivolumab plus Ipilimumab in Advanced Melanoma. N. Engl. J. Med..

[20] Wolchok J.D., Chiarion-Sileni V., Gonzalez R., Rutkowski P., Grob J.J., Cowey C.L., Lao C.D., Wagstaff J., Schadendorf D., Ferrucci P.F. (2017). Overall Survival with Combined Nivolumab and Ipilimumab in Advanced Melanoma. N. Engl. J. Med..

[21] Asher N., Ben-Betzalel G., Lev-Ari S., Shapira-Frommer R., Steinberg-Silman Y., Gochman N., Schachter J., Meirson T., Markel G. (2020). Real world outcomes of ipilimumab and nivolumab in patients with metastatic melanoma. Cancers.

[22] Schulz T.U., Zierold S., Sachse M.M., Pesch G., Tomsitz D., Schilbach K., Kähler K.C., French E.F., Heinzerling L. (2022). Persistent immune-related adverse events after cessation of checkpoint inhibitor therapy: Prevalence and impact on patients’ health-related quality of life. Eur. J. Cancer.

[23] Xing H., Wang Y., Qu B., Wei Q., LI C., Pan C., Li H. (2023). The Current status of steroid-refractory immune-checkpoint-inhibitor-related hepatotoxicity. Transl. Oncol..

[24] Hodi F.S., O’Day S.J., McDermott D.F., Weber R.W., Sosman J.A., Haanen J.B., Gonzalez R., Robert C., Schadendorf D., Hassel J.C. (2010). Improved survival with ipilimumab in patients with metastatic melanoma. N. Engl. J. Med..

[25] Long G.V., Carlino M.S., McNeil C., Ribas A., Gaudy-Marqueste C., Schachter J., Nyakas M., Kee D., Petrella T.M., Blaustein A. (2024). Pembrolizumab versus ipilimumab for advanced melanoma: 10-year follow-up of the phase III KEYNOTE-006 study. Ann. Oncol..

[26] Robert C., Long G.V., Brady B., Dutriaux C., Maio M., Mortier L., Hassel J.C., Rutkowski P., McNeil C., Kalinka-Warzocha E. (2015). Nivolumab in previously untreated melanoma without BRAF mutation. N. Engl. J. Med..

[27] Berghoff A.S., Schur S., Füreder L.M., Gatterbauer B., Dieckmann K., Widhalm G., Hainfellner J., Zielinski C.C., Birner P., Bartsch R. (2016). Descriptive statistical analysis of a real life cohort of 2419 patients with brain metastases of solid cancers. ESMO Open.

[28] Internò V., Sergi M.C., Metta M.E., Guida M., Trerotoli P., Strippoli S., Circelli S., Porta C., Tucci M. (2023). Melanoma Brain Metastases: A Retrospective Analysis of Prognostic Factors and Efficacy of Multimodal Therapies. Cancers.

[29] Tawbi H.A., Hodi F.S., Lipson E.J., Schadendorf D., Ascierto P.A., Matamala L., Castillo Gutiérrez E., Rutkowski P., Gogas H., Lao C.D. (2024). Nivolumab (NIVO) plus relatlimab (RELA) vs NIVO in previously untreated metastatic or unresectable melanoma (RELATIVITY-047): Overall survival (OS) and melanoma-specific survival (MSS) outcomes at 3 years. J. Clin. Oncol..

[30] Robert C., Ribas A., Hamid O., Daud A., Wolchok J.D., Joshua A.M., Hwu W., Weber J.S., Gangadhar C.T., Joseph R.W. (2018). Durable complete response after discontinuation of pembrolizumab in patients with metastatic mela noma. J. Clin. Oncol..

[31] Hamid O., Robert C., Daud A., Hodi F.S., Hwu W.J., Kefford R., Wolchok J.D., Hersey P., Joseph R., Weber J.S. (2019). Five-year survival outcomes for patients with advanced melanoma treated with pembrolizumab in KEYNOTE-001. Ann. Oncol..

[32] Jansen Y.J.L., Rozeman E.A., Mason R., Goldinger S.M., Geukes Foppen S.M., Hoejberg L., Schmidt H., Van Thienen J.F., Haanen J.B.A.G., Tiainen L. (2019). Discontinuation of anti-PD-1 antibody therapy in the absence of disease progression or treatment limiting toxicity: Clinical outcomes in advanced melanoma. Ann. Oncol..

[33] Betof Warner A., Palmer J.S., Shoushtari A.N., Goldman D.A., Panageas S.K., Hayes S.A., Bajwa R., Momtaz P., Callahan M.K., Wolchok J.D. (2020). Long-term outcomes and responses to retreatment in patients with melanoma treated with PD-1 blockade. J. Clin. Oncol..

[34] Valentin J., Ferté T., Dorizy-Vuong V., Dousset L., Prey S., Dutriaux C., Pham-Ledard A., Beylot-Barry M., Gérard E. (2021). Real-world survival in patients with metastatic melanoma after discontinuation of anti-PD-1 immunotherapy for objective response or adverse effects: A retrospective study. J. Oncol..

[35] Rubatto M., Fava P., Stanganelli I., Ribero S., Pigozzo J., Di Giacomo A.M., Ridolfi L., Tronconi M.C., Trojanello C., Bersanelli M. (2023). Discontinuation of anti-PD1 in advanced melanoma: An observational retrospective study from the Italian Melanoma Intergroup. Eur. J. Cancer.

[36] Swami U., Monga V., Bossler A.D., Zakharia Y., Milhem M. (2019). Durable clinical benefit in patients with advanced cutaneous melanoma after discontinuation of anti-PD-1 therapies due to immune-related adverse events. J. Oncol..

[37] Gibney G.T., Zaemes J., Shand S., Shah N.J., Swoboda D., Gardner K., Radfar A., Petronic-Rosic V., Reilly J.M., Al-Refaie W.B. (2021). PET/CT scan and biopsydriven approach for safe anti-PD-1 therapy discontinuation in patients with advanced melanoma. J. Immunother. Cancer.

[38] Schadendorf D., Wolchok J.D., Hodi F.S., Chiarion-Sileni V., Gonzalez R., Rutkowski P., Grob J.J., Cowey C.L., Lao C.D., Chesney J. (2017). Efficacy and Safety Outcomes in Patients With Advanced Melanoma Who Discontinued Treatment with Nivolumab and Ipilimumab Because of Adverse Events: A Pooled Analysis of Randomized Phase II and III Trials. J. Clin. Oncol..

[39] Dimitriou F., Zaremba A., Allayous C., Kähler K.C., Gerard C.L., Festino L., Schäfer S., Toussaint F., Heinzerling F., Hassel J.C. (2021). Sustainable responses in metastatic melanoma patients with and without brain metastases after elective discontinuation of anti-PD1-based immunotherapy due to complete response. Eur. J. Cancer.

[40] Lebbé C., Meyer N., Mortier L., Marquez-Rodas I., Robert C., Rutkowski P., Menzies A., Eigentler T., Ascierto A.P., Smylie M. (2019). Evaluation of Two Dosing Regimens for Nivolumab in Combination with Ipilimumab in Patients With Advanced Melanoma: Results From the Phase IIIb/IV CheckMate 511 Trial. J. Clin. Oncol..

[41] Carlino M.S., Menzies A.M., Atkinson V., Cebon J.S., Jameson M.B., Fitzharris B.M., McNeil C.M., Hill A.G., Ribas A., Atkins M.B. (2020). Long-term Follow-up of Standard-Dose Pembrolizumab Plus Reduced-Dose Ipilimumab in Patients with Advanced Melanoma: KEYNOTE-029 Part 1B. Clin. Cancer Res..

[42] Long G.V., Robert C., Butler M.O., Couture F., Carlino M.S., O’Day S., Atkinson V., Cebon J.S., Brown M.P., Dalle S. (2021). Standard-Dose Pembrolizumab Plus Alternate-Dose Ipilimumab in Advanced Melanoma: KEYNOTE-029 Cohort 1C, a Phase 2 Randomized Study of Two Dosing Schedules. Clin. Cancer Res..

[43] https://ctep.cancer.gov/protocoldevelopment/electronic_applications/docs/ctcae_v5_quick_reference_5x7.pdf.

[44] Seymour L., Bogaerts J., Perrone A., Ford R., Schwartz L.H., Mandrekar S., Lin N.U., Litière S., Dancey J., Chen A. (2017). iRECIST: Guidelines for response criteria for use in trials testing immunotherapeutics. Lancet Oncol..

[45] Tagliaferri L., Lancellotta V., Fionda B., Mangoni M., Casà C., Di Stefani A., Pagliara M.M., D’Aviero A., Schinzari G., Chiesa S. (2022). Immunotherapy and radiotherapy in melanoma: A multidisciplinary comprehensive review. Hum. Vaccin. Immunother..

[46] Rulli E., Legramandi L., Salvati L., Mandala M. (2019). The impact of targeted therapies and immunotherapy in melanoma brain metastasis: A systematic review and meta-analysis. Cancer.

[47] Choong E.S., Lo S., Drummond M., Fogarty G.B., Menzies A.M., Guminski A., Shivalingam B., Clarke K., Long G.V., Hong A.M. (2017). Survival of patients with melanoma brain metastasis treated with stereotactic radiosurgery and active systemic drug therapies. Eur. J. Cancer.

[48] Mamoor M., Postow M.A., Lavery J.A., Baxi S.S., Khan N., Mao J.J., Rogak L.J., Sidlow R., Thom B., Wolchok J.A. (2020). Quality of life in long-term survivors of advanced melanoma treated with checkpoint inhibitors. J. Immunother. Cancer.

[49] Coen O., Corrie P., Marshall H., Plummer R., Ottensmeier C., Hook J., Bell S., Sagoo G.S., Meads D., Bestall J. (2021). The DANTE trial protocol: A randomised phase III trial to evaluate the Duration of Anti-PD-1 monoclonal antibody Treatment in patients with metastatic melanoma. BMC Cancer.

[50] Mulder E.E.A.P., De Joode K., Litière S., Ten Tije A.J., Suijkerbuijk K.P.M., Boers-Sonderen M.J., Hospers G.A.P., de Groot J.W.B., van den Eertwegh A.J.M., Aarts M.J.B. (2021). Early discontinuation of PD-1 blockade upon achieving a complete or partial response in patients with advanced melanoma: The multicentre prospective Safe Stop trial. BMC Cancer.

[51] Janssen J.C., van Dijk B., de Joode K., Aarts M.J.B., Van Den Berkmortel F.W.P.J., Blank C.U., Boers-Sonderen M.J., van den Eertwegh A.J.M., de Groot J.W.B., Jalving M. (2024). Safe Stop IPI-NIVO trial: Early discontinuation of nivolumab upon achieving a complete or partial response in patients with irresectable stage III or metastatic melanoma treated with first-line ipilimumab-nivolumab—Study protocol. BMC Cancer.

